# Supplementary material for: The Effect of Lactiplantibacillus plantarum BX62 Alone or in Combination with Chitosan on the Qualitative Characteristics of Fresh-Cut Apples during Cold Storage
Source: Microorganisms. 2021 Nov 22;9(11):2404. doi: 10.3390/microorganisms9112404 (PMC8618497; doi:10.3390/microorganisms9112404)
Supplement: Supplementary file 1 [file microorganisms-09-02404-s001.zip › microorganisms-1454095-SI.pdf]

Table S1. Correlation between some selected quality parameters of fresh-cut apples in different groups during cold storage

Index	Pearson correlation coefficient (r)			
	Control	CT	BX62	CT + BX62
TA vs. AMB	0.188	−0.250	−0.257	0.118
TA vs. APB	0.183	−0.250	0.436	−0.590
TA vs. YAMs	0.452	−0.250	0.881 *	−0.590
TA vs. LAB	--	--	−0.638	−0.110
TPC vs. AMB	−0.957 *	0.198	−0.070	0.623
TPC vs. APB	−0.562	0.198	−0.286	−0.066
TPC vs. YAMs	−0.548	0.198	−0.551	−0.066
TPC vs. LAB	--	--	0.205	0.456
PPO vs. AMB	−0.671	−0.392	0.379	0.479
PPO vs. APB	−0.360	−0.392	−0.685	−0.112
PPO vs. YAMs	−0.349	−0.392	−0.604	−0.112
PPO vs. LAB	--	--	0.469	0.287

* The value was significant at $P < 0.05$. TA = titratable acidity, TPC = total phenolics content (TPC), PPO = polyphenol oxidase, AMB = aerobic mesophilic bacteria, APB = aerobic psychrophilic bacteria, YAMs = yeast and molds, LAB = lactic acid bacteria.

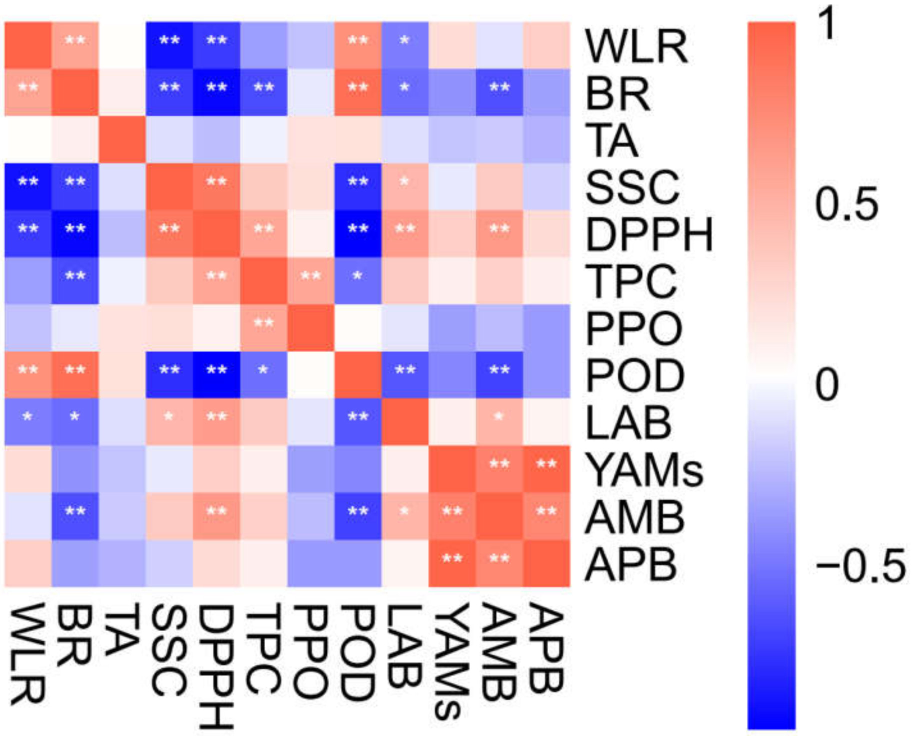

Figure S1. Heat map of correlation between physicochemical and microbial indexes of fresh-cut apples during storage. ** The value was significant at $P < 0.01$, * The value was significant at $P < 0.05$. WLR = weight loss rate; BR = Browning rate; SSC = soluble solids content; POD = peroxidase activity.
